# Grape seed proanthocyanidins ameliorate neuronal oxidative damage by inhibiting GSK-3β-dependent mitochondrial permeability transition pore opening in an experimental model of sporadic Alzheimer’s disease

**DOI:** 10.18632/aging.102041

**Published:** 2019-06-24

**Authors:** Qinru Sun, Ning Jia, Xin Li, Jie Yang, Guomin Chen

**Affiliations:** 1 Institute of Forensic Medicine, Xi’an Jiaotong University Health Science Center, Xi’an, Shaanxi 710061, P.R. China; 2 Department of Human Anatomy, Histology and Embryology, School of Basic Medical Sciences, Xi’an, Jiaotong University Health Science Center, Xi’an Shaanxi 710061, P.R. China; 3 Department of Anesthesiology, First Affiliated Hospital, Xi’an Jiaotong University Health Science Center, Xi’an, Shaanxi 710061, P.R. China

**Keywords:** grape seed proanthocyanidins, glycogen synthase kinase 3β, oxidative stress, mitochondrial permeability transition pore, Alzheimer’s disease

## Abstract

Mitochondria-associated oxidative stress plays a crucial role in Alzheimer’s disease (AD). Grape seed proanthocyanidins (GSPs) have been reported to prevent oxidative stress. In this study, we investigated the underlying mechanisms of GSPs in protecting neurons against oxidative injury in an experimental model of sporadic AD. Primary mouse cortical neurons were subjected to streptozotocin (STZ) to mimic neuronal oxidative damage *in vitro*, and mice were subjected to intracerebroventricular (ICV) injection of STZ as an *in vivo* sporadic AD model. GSPs not only significantly ameliorated neuron loss and mitochondrial dysfunction in mouse cortical neurons pretreated of STZ, but also reduced cognitive impairments, apoptosis and mitochondrial oxidative stress in the cerebral cortex and hippocampus of sporadic AD mice. Moreover, GSPs increased phosphorylation levels of phosphatidylinositol 3-kinase (PI3K), Akt and glycogen synthase kinase 3β (GSK-3β) at its Ser9. Notably, GSPs inhibited STZ-induced mitochondrial permeability transition pore (mPTP) opening via enhancing phosphorylated GSK-3β (p-GSK-3β) binds to adenine nucleotide translocator (ANT), thereby reducing the formation of the complex ANT-cyclophilin D (CypD). In conclusion, GSPs ameliorate neuronal oxidative damage and cognitive impairment by inhibiting GSK-3β-dependent mPTP opening in AD. Our study provides new insights into that GSPs may be a new therapeutic candidate for treatment of AD.

## INTRODUCTION

Alzheimer’s disease (AD) is a neurodegenerative disorder characterized by a progressive decline in cognitive function and an irreversible loss of neurons [1, 2]. AD has become the most prevalent type of dementia, subsequently leading to a major cause of death [3]. In AD brains, the accumulated extracellular senile plaques, consisting predominantly of the amyloid-β (Aβ), and neurofibrillary tangles (NFTs), consisting of hyperphosphorylated tau protein, are neuropathological hallmarks [4]. Both senile plaques and neurofibrillary lesions seem to result in neuronal loss in affected brain areas. However, to date, the therapeutic approaches targeting to Aβ and tau protein have shown limited curative effect in clinical trials. In contrast to the rare and hereditary family AD (FAD), sporadic AD (SAD) is more challenging for relevant medical institutions and researchers in the world [5]. In addition to transgenic animals used to study FAD, rat or mouse subjected to intracerebroventricular (ICV) injections of streptozotocin (STZ) become a feasible and workable model for revealing the mechanisms of SAD or screening medicines [6, 7]. Interestingly, these related studies have suggested that there are some common features between FAD and SAD, such as mitochondrial dysfunction [8] and oxidative stress [9, 10].

Several lines of studies have shown that glycogen synthase kinase 3β (GSK-3β), a ubiquitous serine/threonine kinase abundantly expressed in central neuron system, may appear to function in AD and Huntington’s disease (HD) [11, 12]. There is an increasing number of evidence indicating that GSK-3β is involved in oxidative stress in the relevant brain regions [13–15]. Furthermore, previous studies have suggested SB 216763, an inhibitor of GSK-3β, protects cardiomyocytes from triptolide-induced toxicity by desensitizing mitochondrial permeability transition [16]. A recent study demonstrated that increasing phosphorylation levels of GSK-3β (p-GSK-3β) at its Ser9 could enhance the interaction between adenine nucleotide translocator (ANT) and p-GSK-3β, subsequently inhibiting mitochondrial permeability transition pore (mPTP) opening, reducing mitochondrial superoxide production and preventing neuronal apoptosis [17]. These data indicate that not only does GSK-3β participate in oxidative stress, but it also plays an important role in neuronal apoptosis and cognitive dysfunction. Therefore, it is feasible to improve cognitive function and attenuate neuronal apoptosis by increasing phosphorylation of GSK-3β.

Over the past two decades, increasing evidence has shown that several natural plant extracts possess the effects of anti-inflammation, anti-oxidation and improvement of cognitive functions [18]. Proanthocyanidins are widely distributed in many plants, such as fruits, vegetables, nuts and seeds, especially in grape seeds [19]. Grape seed proanthocyanidins (GSPs), which contain dimers, trimers, oligomers of catechin and epicatechin, have been reported to resist oxidative stress and to promote DNA repair [20, 21]. Grape seed procyanidin B2 prevent podocytes from high glucose-induced mitochondrial dysfunction and apoptotic cell death [22]. In addition, a recent report also confirmed that proanthocyanidins can decrease the apoptotic cell death in hippocampal neurons of a genetic AD mouse model [23]. Taken together, we can assume that p-GSK-3β may mediate neuronal protection of GSPs by inhibiting neuronal oxidative damage, mitochondrial dysfunction and cognitive impairments.

In the present study, primary mouse cortical neurons were treated with STZ *in vitro*, while mice were subjected to ICV injection of STZ as an *in vivo* AD model. Morris Water Maze (MWM) was employed to assess the ability of spatial learning and memory and the rotarod test, a nonvisual-spatial task used to evaluate motor ability and coordination. To investigate the neuroprotective mechanism of GSPs, mitochondrial superoxide production, levels of mitochondrial membrane potential (MMP) and adenosine triphosphate (ATP), the activity of cytochrome c oxidase (CcO), the threshold of mPTP opening and phosphorylation levels of PI3K-Akt-GSK-3β (Ser9) were evaluated *in vitro* and *in vivo*, as well as the levels of the Aβ and phosphorylation of tau (p-tau). Furthermore, in order to elucidate the underlying mechanism, the interaction of p-GSK-3β with ANT and the mPTP opening were determined in primary mouse cortical neurons. This study demonstrates that GSPs ameliorate neuronal oxidative damage and cognitive impairments in an experimental sporadic AD model by inhibiting GSK-3β-dependent mPTP opening. This study provides new insights that GSPs may be a novel therapeutic candidate to treat AD.

## RESULTS

### GSPs attenuated STZ induced neuron loss and apoptosis in primary cortical neurons

**Figure 1:**
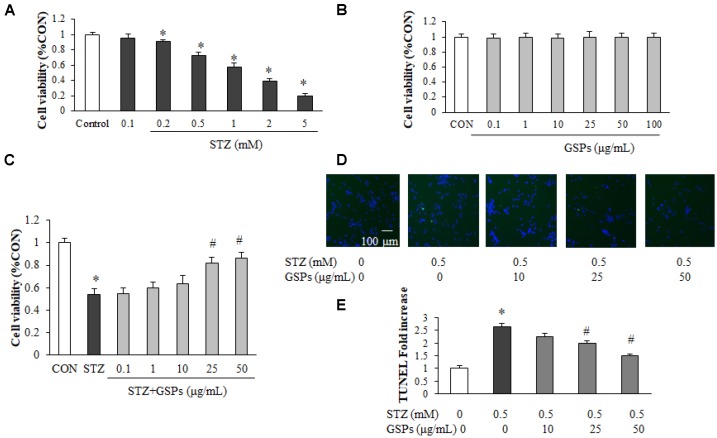
**GSPs alleviated STZ-induced neuron loss and apoptosis in primary mouse cortical neurons.** (**A**, **B**) Neurons were treated with different concentrations of STZ (0.1, 0.2, 0.5, 1, 2 and 5 mM) (**A**) or GSPs (0.1, 1, 10, 20, 50 and 100 μg/mL) for 24 h (B). (**C**) Neurons were pre-incubated with GSPs (0.1, 1, 10, 25 and 50 μg/mL) for 2 h prior to being exposed to 0.5 mM STZ for 24 h. (**D**) Cell apoptosis was measured by TUNEL staining. (scale bar = 100 μm). (**E**) Quantitative analysis of TUNEL staining. ^*^P < 0.05 *vs* CON; ^#^*P* < 0.05 *vs* STZ, n=4.

To assess the neuroprotective effect of GSPs, primary cortical neurons were subjected to (3-(4,5-Dimethylthiazol-2-yl)-2,5-diphenyltetrazolium bromide (MTT) assay. We first examined the neurotoxicity of STZ, and the results showed that STZ with a concentration greater than 0.2 mM caused neuron loss in a dose-dependent manner (Figure 1A). The STZ dose was set to 0.5 mM for primary neuronal cells in all subsequent assays due to a 28.62% ± 4.22% reduction induced by 0.5 mM STZ treatment. As shown in Figure 1B, after treatment with GSPs (0.1 - 100 μg/mL) for 24 h, there was no significant difference of cell viability compared with that in the CON group, indicating that GSPs was nontoxic to primary cortical neurons under the treatment conditions. As demonstrated in Figure 1C, pretreatment with GSPs at the concentrations of 25, 50 μg/mL significantly attenuated cell death in the presence of STZ, whereas the concentrations of 0.1, 1 and 10 μg/mL GSPs did not show obvious effect. To further investigate the neuroprotective effects of GSPs on STZ-induced neuron loss, we evaluated apoptosis by using TdT-mediated dUTP Nick-End Labeling (TUNEL) staining in primary cortical neurons. As shown in Figure 1D and 1E, STZ increased the proportion of TUNEL-positive cells, which was significantly suppressed by pretreated with GSPs at the concentrations of 25 and 50 μg/mL. These results indicate that GSPs can counteract STZ induced neuron loss and apoptosis.

### GSPs ameliorated cognitive impairments in the presence of STZ

**Figure 2:**
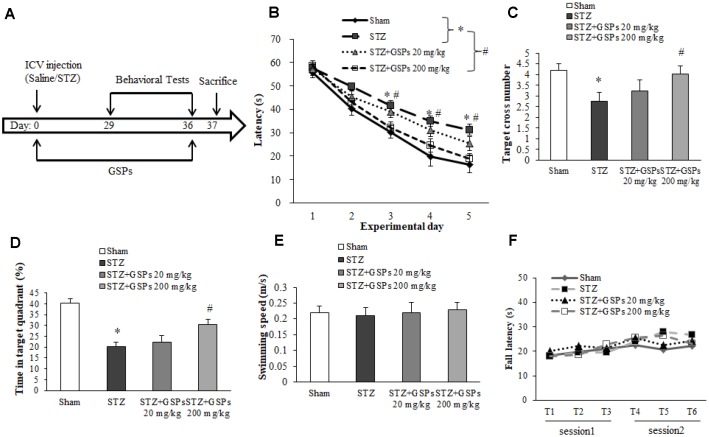
**GSPs effectively counteracted the cognitive impairments in mice subjected to ICV injection of STZ.** (**A**) Schematic diagram of animal study design. (**B**) The escape latency of the training acquisition trials for 5 consecutive days in Morris water maze (MWM). (**C**) The target cross number of the probe test in MWM. (**D**) The time in target quadrant in the probe test in MWM. (**E**) The swimming speed in the probe test in MWM. (**F**) The fall latency in the rotarod test. ^*^P < 0.05 *vs* Sham; ^#^*P* < 0.05 *vs* STZ, n=10.

The schematic diagram of animal study design is shown in Figure 2A. To investigate the effectiveness of GSPs against STZ-induced neurotoxicity *in vivo*, we performed Morris water maze (MWM). The results showed that the escape latency in the STZ group was significantly much longer than that in the Sham group on the 3^rd^, 4^th^ and 5^th^ experiment day respectively (Figure 2B). In the probe test, the target cross number and the time in target quadrant in the STZ group were obviously decreased compared with that in the Sham group (Figure 2C and 2D). Administration of high dosage of GSPs (200 mg/kg) significantly counteracted the effect of STZ on the escape latency, the target cross number and the time in target quadrant, while the low dosage of GSPs (20 mg/kg) showed no significant changes (Figure 2B–2D). There was no significant difference in the swimming speeds of mice from different groups (Figure 2E). These data indicate that GSPs are beneficial for learning and memory in the presence of STZ.

To test the motor coordination and balance, mice were examined by using the rotarod test. The results showed that there is no significant difference in the fall latency between the mice with ICV injection of STZ and the mice from the sham group (Figure 2F). Similarly, administration of different concentration of GSPs with pretreatment with ICV injection of STZ did not affect the fall latency (Figure 2F). These data indicate that STZ does not impair motor coordinator or balance, while GSPs did not affect the motor activity.

### GSPs attenuated STZ-induced Aβ production and tau phosphorylation

**Figure 3:**
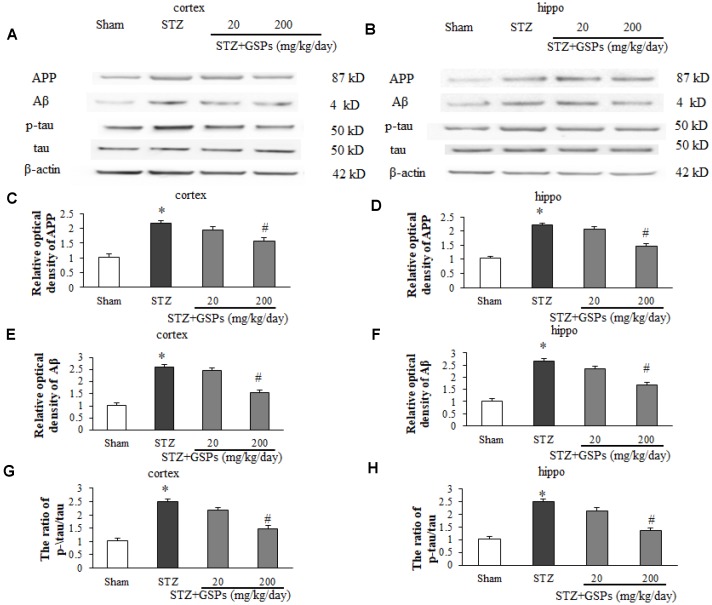
**GSPs inhibited STZ-induced Aβ accumulation and phosphorylation of tau *in vivo*.** (**A** and **B**) Representative immunoblot bands for APP, Aβ, p-tau and tau in the cerebral cortex and hippocampus, respectively. (**C**–**F**) Quantification analysis of immunoblot bands. Protein expression levels were normalized to β-actin. ^*^P < 0.05 *vs* Sham; ^#^*P* < 0.05 *vs* STZ, n=4-6.

In this study, alongside the cognitive ability, we investigated the effects of STZ on alterations of Aβ production and tau phosphorylation in cerebral cortex and hippocampus. The results showed that ICV injection of STZ significantly increased the expression levels of amyloid precursor protein (APP) and Aβ in the cerebral cortex and hippocampus (Figure 3A–3F). In addition, ICV injection of STZ significantly increased the level of phosphorylated tau (p-tau) in the cerebral cortex and hippocampus, although there is no significant difference in the level of tau (Figure 3A, 3B, 3G and 3H). Interestingly, GSPs (200 mg/kg) significantly reduced Aβ production and tau phosphorylation induced by STZ (Figure 3A–3H), coinciding with its protection of cognitive functions. These results indicate that GSPs play an important role in blocking Aβ production and tau phosphorylation.

### GSPs counteracted neuron loss induced by STZ in mouse cerebral cortex and hippocampus

**Figure 4:**
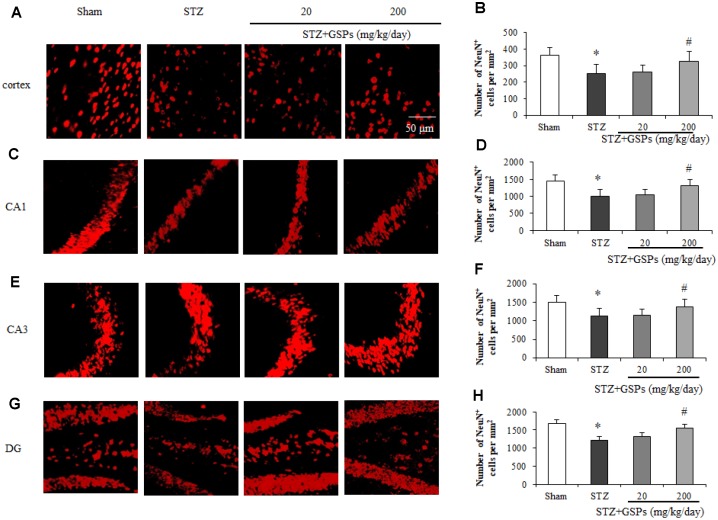
**GSPs eliminated STZ-induced neuronal loss in the cortex and CA1, CA3 and DG of hippocampus.** Representative images of immunofluorescence staining assay of NeuN in the cortex (**A**), hippocampal CA1 (**C**), CA3 (**E**) and dentate gyrus (**G**) areas. Quantification of NeuN-positive cells in the cortex (**B**), hippocampal CA1 (**D**), CA3 (**F**) and dentate gyrus (**H**) areas. ^*^P < 0.05, ^**^P < 0.01 *vs* Sham; ^#^*P* < 0.05, ^#^*P* < 0.01 *vs* STZ, n=4-6.

To investigate the effects of STZ on the number of neurons in mouse cerebral cortex and hippocampus, immunofluorescence staining of NeuN, a marker of the nucleus of neurons, was conducted. As shown in Figure 4A–4H, the nucleus of stained neurons in the cerebral cortex and hippocampus displayed red. ICV injection of STZ significantly reduced the number of NeuN-positive cells in the cerebral cortex and hippocampal CA1, CA3 and dentate gyrus areas. However, administration of GSPs (200 mg/kg) significantly reversed the decrease of the number of neurons in the cerebral cortex and various areas of hippocampus induced by STZ. These results indicate that GSPs ameliorate neurons loss induced by STZ in the cerebral cortex and hippocampus.

### GSPs counteracted apoptotic cell death induced by STZ in mouse cerebral cortex and hippocampus

**Figure 5:**
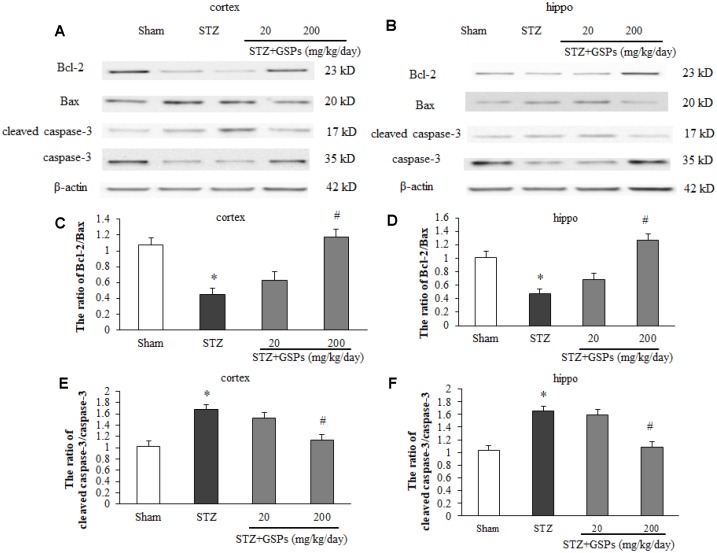
**GSPs inhibited STZ-induced mitochondria-associated apoptosis signaling pathway *in vivo*.** (**A** and **B**), Representative immunoblot bands for Bcl-2, Bax, cleaved caspase-3 and caspase-3 in the cerebral cortex and hippocampus. (**C-F**), Quantification analysis of immunoblot bands. Protein expression levels were normalized to β-actin. ^*^P < 0.05 *vs* Sham; ^#^*P* < 0.05 *vs* STZ, n=4-6.

Given the important role of apoptosis in STZ-induced cognitive impairments *in vivo*, we next examined the levels of apoptosis-associated proteins in the mouse cerebral cortex and hippocampus. As shown in Figure 5A and 5B, the expression levels of cleaved-caspase 3, Bcl-2 and Bax were determined by employing Western blotting. The expression of Bcl-2 was decreased, and the levels of Bax and cleaved-caspase 3 were significantly increased in mice cerebral cortex and hippocampus in the STZ group (Figure 5C–5F). Administration of high dosage of GSPs not only reversed the expression level of Bcl-2 in the cerebral cortex and hippocampus, but also significantly abated the levels of Bax and cleaved-caspase 3 increased by STZ (Figure 5A–5F). The low dosage of GSPs did not show obvious protection against apoptotic cell death induced by STZ. These results indicate that GSPs can abolish neuronal apoptosis induced by STZ in the cerebral cortex and hippocampus of mouse.

### GSPs improved mitochondrial function in the presence of STZ *in vitro* and *in vivo*

**Figure 6:**
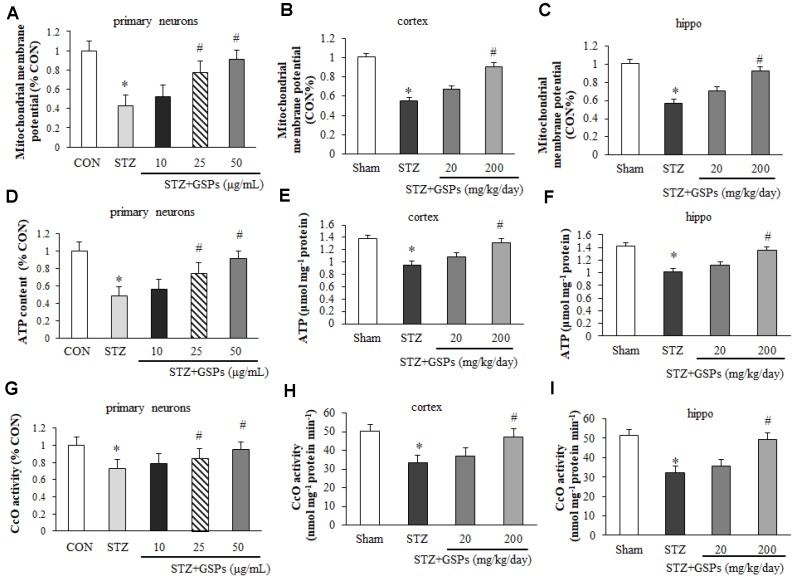
**GSPs improved the levels of mitochondrial membrane potential (MMP), ATP and the activity of CcO *in vitro* and *in vivo*.** Pretreatment with 25 ug/mL or 50 ug/mL GSPs significantly increased STZ decreased levels of MMP (**A**), ATP (**D**) and the activity of CcO (**G**) in primary cortical neurons. Administration of high dosage of GSPs significantly increased STZ decreased levels of MMP (**B**), ATP (**E**) and the activity of CcO (**H**) in the cerebral cortex. Intake of high dosage of GSPs significantly increased levels of MMP (**C**), ATP level (**F**) and the activity of CcO (**I**) in the hippocampus. ^*^ *P* < 0.01 *vs* CON or Sham; ^#^ *P* < 0.01 *vs* STZ, n=4-6.

In order to assess the effects of GSPs on mitochondrial function, we examined the levels of MMP, cellular ATP and the activity of CcO *in vitro* and *in vivo*. As shown in Figure 6A, a significant decrease in the fluorescent signal of rhodamine 123 was observed in primary cortical neurons exposed to STZ, indicating a significantly loss of MMP. However, an obvious increase in MMP was observed in cells pretreated with GSPs. Similarly, our results showed that the level of ATP (Figure 6D) and the activity of CcO (Figure 6G) were decreased in the presence of STZ, whereas this effect was reversed by administration of 200 mg/kg GSPs. Similarly, the levels of MMP (Figure 6B and 6C), cellular ATP (Figure 6E and 6F) and the activity of CcO (Figure 6H and 6I) were decreased in the cerebral cortex and hippocampus of the mice treated with STZ. In addition, the level of MMP was significantly decreased in the STZ group compared with that in the Sham group, whereas high dosage of GSPs significantly improved the level of MMP (Figure 6B and 6C). Administration of high dosage of GSPs significantly prevented the decrease of the ATP level (Figure 6E and 6F) and the activity of CcO (Figure 6H and 6I) in cerebral cortex and hippocampus of the mice treated with STZ as well. Taken together, STZ significantly decreases the levels of MMP, ATP and the activity of CcO, which could be rescued by administration of GSPs.

### GSPs reduced mitochondrial superoxide production and mPTP opening after ICV injection of STZ in the cerebral cortex and hippocampus

**Figure 7:**
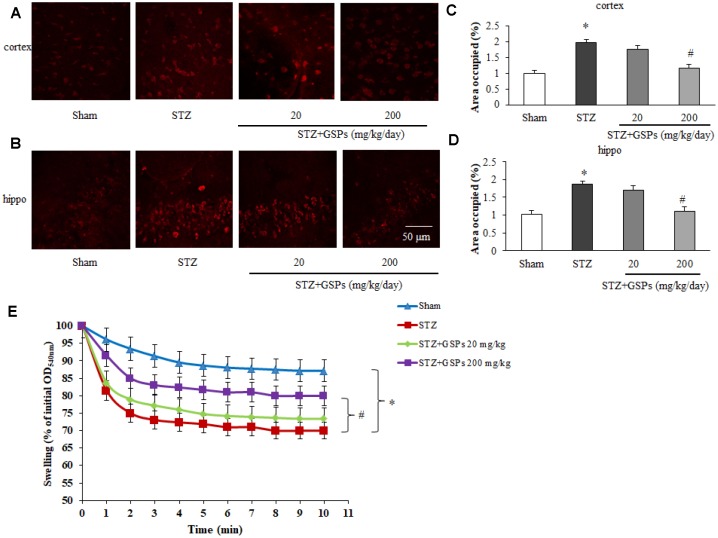
**GSPs blocked STZ-induced mitochondrial superoxide production and mitochondrial swelling.** (**A** and **B**) Representative fluorescent staining of MitoSOX Red in the cerebral cortex and the hippocampus CA1 region of mice. (**C** and **D**) Quantification analysis showed changes of area occupied by fluorescent staining. (**E**) Comparison of the mitochondrial swelling among different groups. ^*^ *P* < 0.01 *vs* Sham; ^#^ *P* < 0.01 *vs* STZ, n=6.

To further investigate a possible involvement of the mitochondrial superoxide production with STZ-induced mitochondrial dysfunction, we manipulated the MitoSOX Red staining in mice brain slices. As shown in Figure 7A–7D, we found a significant increase in mitochondrial superoxide production in mouse cerebral cortex and hippocampus after ICV injection of STZ. Administration of high dosage of GSPs obviously reduced the mitochondrial superoxide production.

The opening of mPTP causes mitochondrial swelling, release of pro-apoptotic proteins and uncoupling of mitochondrial oxidative phosphorylation. To validate the effects of GSPs on the threshold of mPTP opening, mitochondrial swelling assay was applied. The results showed that the absorbance at optical density 540 nm (OD_540_nm) was significantly decreased in STZ-treated mice, indicating a decrease of the threshold of mPTP opening. However, the threshold of mPTP opening was partly restored by high dosage of GSPs after ICV injection of STZ (Figure 7E).

These results indicate that GSPs can prevent STZ-induced mitochondrial superoxide generation and mPTP opening in the cerebral cortex and hippocampus.

### GSPs enhanced phosphorylation of PI3K-Akt-GSK-3β (Ser9) pathway *in vitro* and *in vivo*

**Figure 8:**
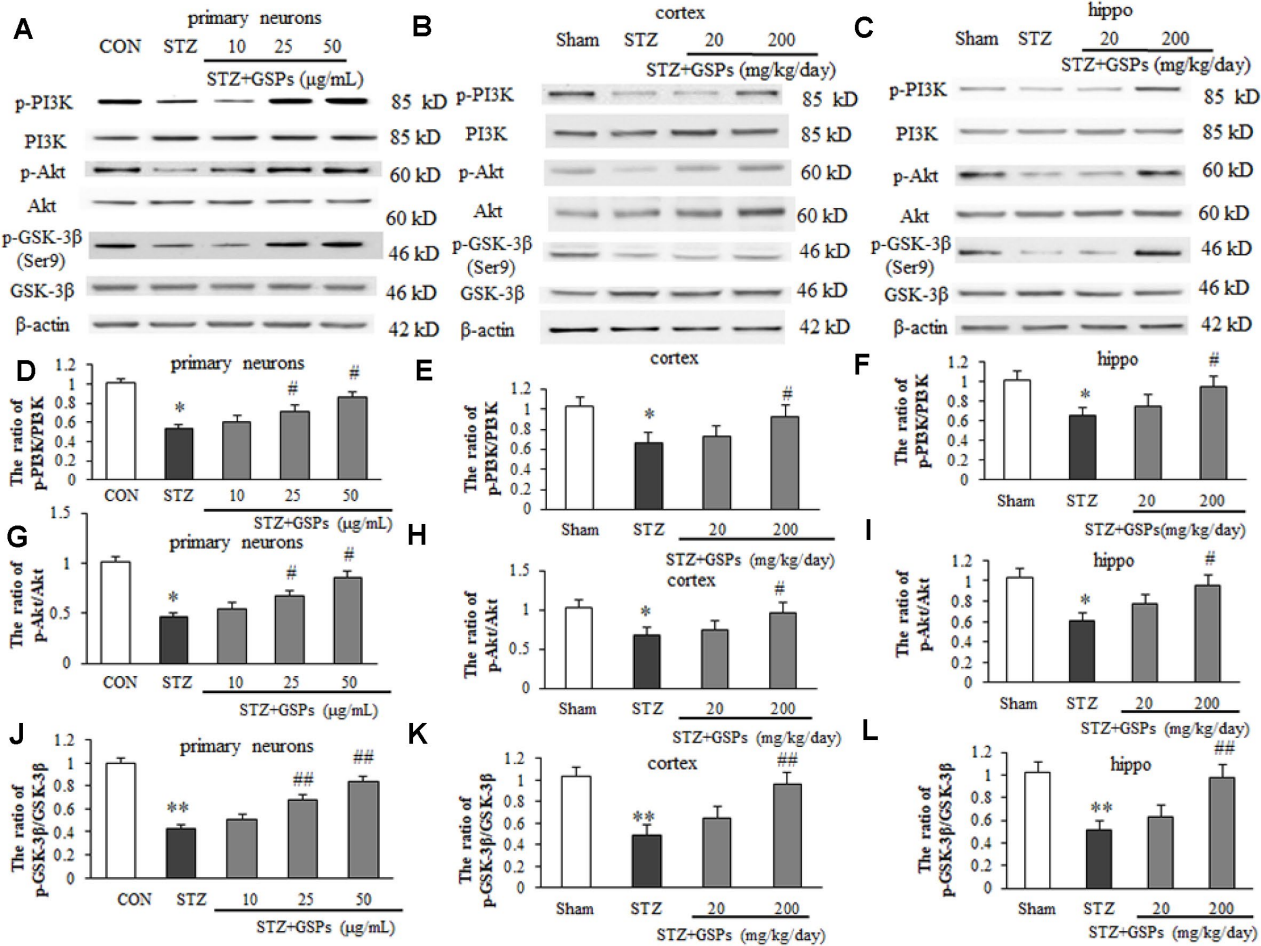
**GSPs improved STZ-reduced ratios of p-PI3K/ PI3K, p-Akt/Akt and p- GSK-3β/GSK-3β *in vitro* and *in vivo*.** (**A**–**C**) Representative immunoblot bands for p-PI3K, PI3K, p-Akt, Akt, p-GSK-3β (Ser9), GSK-3β and β-actin in primary cortical neurons, the cerebral cortex and hippocampus, respectively. (**D**–**L**) Quantification analysis of immunoblot bands. Protein expression levels were normalized to β-actin. ^*^P < 0.05, ^**^P < 0.01 *vs* CON or Sham; ^#^*P* < 0.05, ^##^*P* < 0.01 *vs* STZ, n=4-6.

Phosphorylation of GSK-3β at its Ser9 is thought to be involved in inhibiting mPTP opening, which is positively regulated by the PI3K-Akt pathway. In this study, the phosphorylation levels of PI3K, Akt and GSK-3β (Ser9) were further evaluated by Western blotting *in vitro* and *in vivo*. As shown in Figure 8A, STZ induced a significant decrease in phosphorylation levels of PI3K, Akt and GSK-3β (Ser9) in primary cortical neurons. However, pretreatment with GSPs of 25 and 50 μg/mL obviously increased the phosphorylation levels of PI3K, Akt and GSK-3β (Ser9) in primary cortical neurons (Figure 8D, 8G and 8J). We further confirmed that GSPs counteracted STZ-induced decrease of phosphorylation levels of PI3K, Akt and GSK-3β (Ser9) in mouse cerebral cortex and hippocampus (Figure 8B and 8C). As shown in Figure 8E, 8F, 8H and 8I, the ratio of p-PI3K/PI3K and p-Akt/Akt were decreased in the mouse cerebral cortex and hippocampus of STZ group. Notably, STZ significantly reduced the level of p-GSK-3β (Ser9) (Figure 8B and 8C). High dosage of GSPs significantly enhanced the ratios of p-PI3K/PI3K and p-Akt/Akt (Figure 8E, 8F, 8H and 8I). As expected, phosphorylation of GSK-3β at its Ser9 was enhanced by administration of high dosage of GSPs (Figure 8K and 8L). These results indicate that GSPs act as an activator of p-GSK-3β (Ser9) in neurons.

### GSPs attenuated STZ-induced mPTP opening by activating PI3K-Akt-dependent phosphorylation of GSK-3β (Ser9)

**Figure 9:**
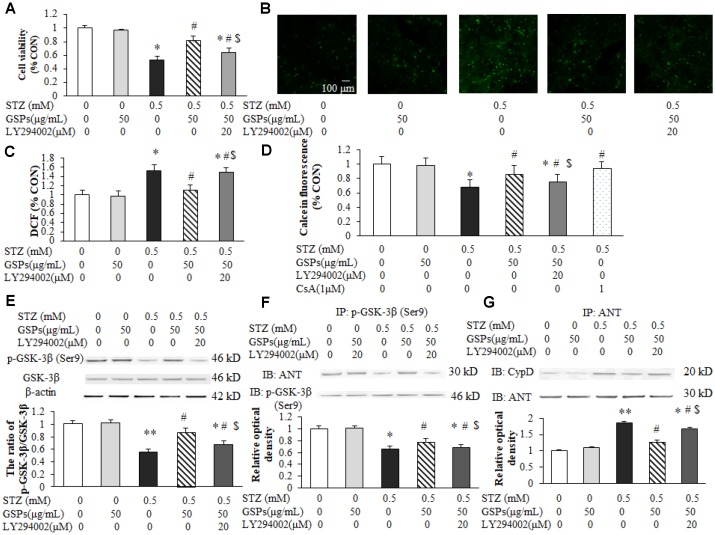
**GSPs attenuated STZ-induced mPTP opening by enhancing PI3K-Akt-dependent phosphorylation of GSK-3β (Ser9) in primary cortical neurons.** (**A**) Neurons were co-pretreated with of 50 μg/mL GSPs and LY294002 (20 μM) for 2 h and then treated with STZ (0.5 mM) for 24 h. The cell viability was detected by using MTT assay and is represented as the percentage of the CON (non-treated) cells. (**B**) The representative images of H_2_DCFDA staining (green) in primary cortical neurons. (**C**) The quantification analysis of the H_2_DCFDA staining among different groups. (**D**) GSPs inhibited STZ-induced mPTP opening, which was blocked by LY294002. (**E**) GSPs increased phosphorylation of GSK-3β at Ser9 in the presence of STZ, which was blocked by LY294002. (**F**) GSPs increased the binding of p-GSK-3β and ANT in the presence of STZ, and LY294002 inhibited the effects of GSPs. (**G**) Immunoprecipitation experiments showed that GSPs reduced STZ-induced binding of ANT to CypD, which was blocked by LY294002. ^*^P < 0.05, ^**^P < 0.01 *vs* CON; ^#^*P* < 0.05 *vs* STZ; ^$^*P* < 0.05 *vs* STZ+GSPs; n=4.

To evaluate whether phosphorylation of GSK-3β (Ser9) participated in the neuroprotective effect of GSPs, LY294002 was co-incubated with GSPs in primary cortical neurons in the presence of STZ. MTT assay results showed that co-treatment with LY294002 markedly blocked the neuroprotection of GSPs against STZ (Figure 9A). We next determined the reactive oxygen species (ROS) production in neurons exposed to STZ. Consistently, as shown in Figure 9B and 9C, an increase of intracellular ROS production was evident after treatment of STZ, which was attenuated by pretreated with GSPs. LY294002 preserved intracellular ROS production when co-cultured with GSPs in the presence of STZ. These results indicate that GSPs can counteract STZ-induced neuron loss and oxidative stress in primary cortical neurons by activating the PI3K pathway. To further explore whether GSK-3β dependent mPTP opening mediated the neuroprotection of GSPs, Calcein/CoCl_2_ staining was conducted. As shown in Figure 9D, STZ significantly decreased the intensity of Calcein-AM, which was partially prevented by pre-treatment with GSPs. However, LY294002 significantly blocked this enhancement of intensity of Calcein-AM. Furthermore, GSPs increased phosphorylation of GSK-3β at its Ser9 in the present of STZ, which was blocked by LY294002 (Figure 9E).

Previous studies have shown that mPTP is modulated by cyclophilin D (CypD) or adenine nucleotide translocator (ANT) [24]. As shown in Figure 9F and 9G, STZ significantly decreased the interaction between p-GSK-3β (Ser9) and ANT, and increased generation of the ANT/CypD complex. However, pretreatment of GSPs decreased the binding of ANT to CypD by promoting the interaction of p-GSK-3β (Ser9) and ANT, which was significantly blocked by LY294002. These results suggest that GSPs reduce GSK-3β-dependent mPTP opening by enhancing GSK-3β phosphorylation at its Ser9.

## DISCUSSION

Numerous studies have indicated that AD is the most prevalent neurodegeneration disorder with irreversible progressive cognitive impairments. However, due to the complexity of AD pathogenesis, curing and preventing AD still face serious challenges. Focusing on pathogenesis research of AD and development of effective prevention measures and drugs has important clinical value and practical significance. The main findings of the present study indicate that GSPs ameliorate neuronal oxidative damage and cognitive impairments in AD by inhibiting GSK-3β-dependent mPTP opening.

Previous studies showed that neurons exposed to STZ displayed AD-like pathology, such as amyloid angiopathy [25], cognitive impairments [26], oxidative stress [4], and mitochondrial dysfunction [27]. In our study, to mimic neuron damage in AD, the primary mouse cortical neurons were treated with STZ, and mice were subjected to ICV injection of STZ. The results showed that primary mouse cortical neurons treated with STZ displayed obvious neuron loss and apoptosis, while the mice with ICV injection of STZ showed significant cognitive dysfunction in Morris water maze. In addition, ICV injection of STZ did not affect motor coordination and balance of mice assessed by using a rotarod test. These results demonstrate that we successfully set up STZ-induced AD-like neuron damage *in vitro* and *in vivo*. Although GSPs are considered to be beneficial for the therapy of patients with cardiovascular diseases and metabolic diseases [28], the knowledge about GSPs against AD is still fragmentary. In the present study, GSPs protected against STZ-induced neuron loss *in vitro*, as shown by decreasing cell apoptosis death, and *in vivo,* GSPs also alleviated STZ-induced cognition impairments in Morris water maze. Our results indicate that GSPs can improve cognition and protect neuronal cells against neurotoxicity of STZ.

The major neuropathologic hallmarks of AD include intracellular hyperphosphorylated neurofibrillary tau tangles (NFTs), extracellular deposits of plaques of amyloid β (Aβ) [4]. In this study, we found that ICV injection of STZ increased the expression levels of APP, Aβ and p-tau in the cerebral cortex and hippocampus. These results are consistent with the previous studies [20, 29]. As expected, administration of 200 mg/kg GSPs decreased the levels of APP, Aβ and p-tau in the presence of STZ, indicating that GSPs is effective against Aβ accumulation and tau phosphorylation induced by STZ.

In addition to the formation of senile plaques-containing Aβ, NFTs, loss of neurons and synaptic dysfunction in affected brain areas are common pathological changes in FAD and SAD. Neuronal loss in the affected brain areas is thought to be associated with synaptic loss and impairment of learning and memory abilities. In this study, STZ significantly boosted neuronal apoptosis in the hippocampus and cerebral cortex. However, administration of sufficient GSPs mitigated STZ-induced apoptosis. Our results demonstrate that GSPs can prevent neuronal apoptosis induced by STZ, which is consistent with the neuroprotection of GSPs in a genetic AD mice model [23].

Prior to other cellular alterations, mitochondrial damage is the typical manifestation during the process of mitochondria-mediated apoptosis. In addition, the opening of mPTP is increased under oxidative stress conditions, adenine nucleotide depletion or membrane depolarization [30]. Once the opening of mPTP is triggered, it allows molecules <1.5 kDa to pass through the mitochondrial membrane, causing uncoupling of the electron respiratory chain, mitochondrial depolarization and mitochondrial membrane potential (MMP) dissipation, followed by progressive mitochondrial swelling and rupture of mitochondrial outer membrane. Finally, leakage of pro-apoptotic factors from mitochondria stimulates caspase activation, which ultimately leads to cell death [31]. In this study, the results showed that STZ significantly impaired mitochondrial function *in vitro* and *in vivo.* However, GSPs significantly prevented STZ-induced mitochondrial dysfunction, reactive oxygen species (ROS) production and increased threshold of mPTP opening. These results suggest that GSPs rescue mitochondrial function and prevent excessive ROS production, which might be an important step in interrupting consequent neuronal apoptosis. It has been shown that grape seed proanthocyanidins extract reverse the hippocampal dysfunction by reducing oxidative stress and preserving mitochondrial function in a rat chronic seizures model [32]. Taken together, our study provides empirical evidence to support the theory of neuronal protection of GSPs by rescuing mitochondrial function in AD mice.

GSK-3β, an isoform of GSK-3, is involved in regulating energy metabolism and mitochondria-associated apoptotic cell death. Previous studies have shown that GSK-3β is negatively regulated by its upstream signaling pathway PI3K/Akt, which plays a key role in diverse cellular activities and promotes cell growth and survival [33]. In this study, we investigated whether GSK-3β was involved in GSPs-resisted apoptosis in the presence of STZ. The results showed that STZ reduced the phosphorylation levels of PI3K-Akt-GSK3β pathway proteins, whereas GSPs enhanced the phosphorylation levels of these proteins. These results suggest that the neuronal protection of GSPs against STZ is mediated by the activation of PI3K-Akt-dependent GSK-3β inactivation, phosphorylated GSK-3β at its Ser9.

mPTP opening is modulated by cellular signaling cascades, involving binding of pro-apoptotic and anti-apoptotic proteins to CypD or adenine nucleotide translocator (ANT) [34]. Diverse proteins, such as transcription factors, kinases and signaling proteins exhibit the ability to interact with ANT [35]. A recent study suggests that p-GSK-3β (Ser9) inhibits mPTP opening by regulating the binding of ANT to cyclophilin D (CypD) [17]. In the present study, STZ significantly reduced cell viability and increased intracellular ROS production, which were attenuated by pretreatment with GSPs. In addition, GSPs increased the phosphorylation of PI3K-Akt-GSK-3β (Ser9) in the presence of STZ. Notably, GSPs increased the binding of p-GSK-3β (Ser9) to ANT and reduced the formation of ANT-CypD complexes, which subsequently increased the threshold of mPTP opening. More importantly, co-pretreatment with LY294002 (an inhibitor of PI3K) abolished the anti-apoptotic and anti-oxidative effects of GSPs in the presence of STZ. LY294002 also decreased GSPs-enhanced phosphorylation of PI3k-Akt and GSK-3β at its Ser9 site. Furthermore, LY294002 decreased GSPs-increased threshold of mPTP opening by reducing the binding between p-GSK-3β (Ser9) and ANT. All the above data reveal that GSPs inhibit mPTP opening by activation of the PI3K-Akt pathway, which increases the phosphorylation level of GSK-3β at its Ser9, thereby disturbing the formation of ANT-CypD complex.

In conclusion, GSPs could counteract neuronal loss and cognitive impairments induced by STZ. In addition, GSPs mitigates STZ-induced neuronal apoptosis, excessive ROS generation and mitochondrial dysfunction. Notably, GSPs inhibited mPTP opening by enhancing GSK-3β phosphorylation at its Ser9. The present study provides a new theoretical foundation for GSPs as a potential medication to prevent occurrence of cognitive impairments in AD.

## MATERIALS AND METHODS

### Animals and surgery

For ICV injection of STZ, different strains of mice were used including C57BL/6 mice, Swiss albino mice and Kunming mice. After ICV injection, the cognitive function of different strains of mice was decreased [20, 36–39]. In the present study, male C57BL/6 mice (25 - 30 g, 8 weeks old) were allowed access to food and water *ad libitum*, and maintained at 23 ± 1 °C under a 12:12 h light/dark cycle. All procedures were approved by the Institutional Animals Care and Use Committee at Xi’an Jiaotong University (XJTULAC-2015-328) and measurements were taken to minimize discomfort in accordance with the National Institutes of Health Guide for Care and Use of Laboratory.

C57BL/6 mice were subjected to stereotaxic apparatus for injection of STZ (Sigma, USA) into the left lateral ventricle of mice as previously described [20]. For ICV injection, briefly, mice were anesthetized by using chloral hydrate (0.35 g/kg). The mouse’s head was restrained onto a stereotaxic apparatus. To locate the bregma, the scalp was incised. The bregma coordinates used were -1.0 mm lateral, -0.3 mm posterior, and -2.5 mm below. Each mouse received a single ICV injection of 3.0 mg/kg STZ in 3.0 μL saline into the left ventricle of the brain.

### Cortical neuron culture

Primary cortical neurons were cultured as described previously with minor modification. Briefly, cortical tissues were dissected from newborn (P0 to P1) pups of C57BL/6 mice, dissociated with 0.05% trypsin, and triturated in ice-cold Neurobasal A medium. The mixtures were centrifuged at 200 x *g* for 5 min. Then, the pellet was resuspended in culture medium (Neurobasal A medium with 2% B27 supplement, 0.5 mM L-glutamine, 100 U/mL penicillin and 100 μg/mL streptomycin). Neurons were seeded onto poly-L-lysine coated culture plates and maintained in at 37 °C in 5% CO_2_ incubator.

### Dosage information / Dosage regimen

GSPs were purchased from Tianjin Jianfeng Natural Product R&D Co., Ltd in China. GSPs contain 98.20% total polyphenols, including 85.22% oligomeric proanthocyanidins, 6.99% catechin, 4.17% epicatechin. GSPs were freshly prepared in drinking water every morning. After ICV injection of STZ, mice were administrated with GSPs for 28 days. Two dosages of GSPs (20 mg/kg/day, 200 mg/kg/day) were given to mice according to previous study with minor modifications [40]. The human equivalent dose (HED) was 1.6 mg/kg or 16 mg/kg, respectively. All animals were divided into 4 groups: the mice injected with saline in lateral ventricle constituted the sham operation group (Sham); the mice receiving ICV injection of STZ in lateral ventricle constituted the STZ group; the mice administered with low dosage of GSPs after ICV injection of STZ constituted the GSPs 20 mg/kg group; the mice administered with high dosage of GSPs after ICV injection of STZ constituted the GSPs 200 mg/kg group.

For *in vitro* study, GSPs were dissolved in the medium and stock solutions for different final concentration (0.5, 1, 10, 25, 50, 100 μg/mL) were prepared. LY294002 (Sigma, USA), an inhibitor of PI3K, was added into the culture medium to a final concentration of 20 μM. On day 7 of DIV, neurons were treated with one or more of the above reagents and divided into 5 groups according to the various treatments: CON group (non-treatment), STZ group (STZ treatment for 24 h), GSPs + STZ group (pretreatment with GSPs for 2 h before STZ treatment) and LY294002 + GSPs + STZ group (pretreatment with LY294002 and GSPs for 2 h before STZ treatment). All experiments were carried out after incubation with STZ for 24 h.

### Morris water maze

The Morris Water Maze was employed to test spatial learning and memory, and the protocol was modified as previously reported in methods [41]. Briefly, water was filled in a circular pool (150 cm diameter, 30 cm high) at 21-23 °C. A hidden platform (10 cm in diameter) was placed 0.5 cm below the surface of water. The latency and the target cross number were recorded by using a computerized video imaging analysis system (Morris Water-maze Tracking System-100, Chengdu Taimeng software Co. Ltd., China). Each mouse was trained three times a day for five consecutive days, and the starting point was randomly changed in the experiment. For each trial, each mouse was allowed to swim for a maximum of 60 sec. The probe test was performed without the hidden platform. Each mouse was placed in the water and allowed to swim for 60 sec freely. The number of platform crossing times was recorded as the target cross number.

### Rotarod test

To detect motor coordination and balance, mice were subjected to the rotarod test. In brief, mice were individually placed on a cylinder where the speed was pre-assigned from 4 to 40 rpm every 5-min period. Each mouse was tested for three trials in two sessions and the inter-trial interval of each trial was 15 min. The fall latency was recorded and calculated. All mice were tested within the same experiment on the same day.

### Immunohistofluorescence staining

Immunohistochemical staining was performed as previously described [42]. Briefly, the brain was removed and fixed by 4% paraformaldehyde. By using a frozen microtome, the brain was cut into 20 μm sections. The free-floating sections were incubated with 0.5% Triton X-100 for 30 min and immersed in 10% normal goat serum in phosphate-buffered saline (PBS) for 30 min. Sections were incubated with rabbit anti-NeuN antibody at 4 °C, and then incubated with fluorescence-conjugated secondary antibody (Tetramethylrhodamine, TRITC) at room temperature. Images were captured by using Zeiss Axio Scope A1 microscope. Three fields of section (100× magnification) randomly placed were photographed and the number of Neun-positively cells were counted.

### Mitochondrial swelling assay

Brain mitochondria were prepared as previously described [43]. The appropriate amount of mitochondria was determined for mitochondrial swelling in 1 mL of mitochondrial isolation buffer. Mitochondrial swelling was triggered by the addition of calcium (200 μM). Swelling was observed by immediately and continuously recording changes at OD_540_ nm by using a spectrophotometer at 10 sec intervals for a total of 600 sec.

### Measurement of mitochondrial superoxide *in vivo*

To measure the mitochondrial superoxide production *in vivo*, mouse brain was removed rapidly and placed on an ice-cold box. The brain was cut into 30 μm thick sections, and each section was loaded with MitoSOX Red (5 μM, Invitrogen) for 30 min [44–46]. The fluorescence of the oxidized dye in the sections was detected by using a laser confocal microscope (TCS-SP2, Germany). The excitation and emission wavelengths were 498 and 522 nm, respectively. ROS fluorescence values were analyzed using the Leica SP2 software. To avoid auto-oxidation, photography was usually finished quickly.

### Measurement of cell viability assay and mitochondrial function

Cell viability was measured by using the MTT assay kit. Mitochondrial membrane potential, ATP levels and activity of cytochrome c oxidase were measured by using the commercial assay kits respectively as previously described [41].

### Measurement of intracellular ROS

Intracellular ROS levels were monitored by using the 2’,7’-Dichlorofluorescin diacetate (H_2_DCFDA) fluorescent probe (Invitrogen). Briefly, neurons were incubated with 10 μM H_2_DCFDA for 30 min at 37 °C. After washing twice with PBS, the fluorescence intensity was observed by using a confocal microscopy. The intensity of fluorescence staining was analyzed with Image J software (NIH).

### Calcein/CoCl_2_ staining

According to the manufacturer’s instructions, the Calcein/CoCl_2_ staining was detected by using a Calcein staining Assay Kit (Invitrogen). Briefly, after treatments, neurons were loaded with 1 μM Calcein-AM (green), 2 mM CoCl_2_ and 20 nM Mitotracker Red at 37 °C for 20 min in phenol red free Hank's buffered salt solution. After washing, the cells were imaged by using a confocal microscopy. The mPTP inhibitor, cyclosporin A (CsA) (1 μM, LC laboratories) was applied as a positive control.

### Western blotting and immunoprecipitation

Protein was extracted from mouse brain or cortical neurons. Protein samples were separated by SDS-PAGE and transferred to Polyvinylidene Fluoride (PVDF) membranes. The membrane was probed with following primary antibodies: polyclonal mouse anti β-actin (Sigma); mouse anti-GSK-3β, rabbit anti-p-GSK-3β (ser9), rabbit anti-p-Akt, mouse anti-Akt, mouse anti-p-PI3K, rabbit anti-PI3K, monoclonal mouse anti-Bax, monoclonal mouse anti-Bcl-2 (Cell Signaling Technology); polyclonal rabbit anti-caspase-3, goat anti-ANT (Santa Cruz); mouse anti-CypD (Abcam), mouse anti-tau (Cell Signaling Technology), anti-p-tau (Cell Signaling Technology), rabbit anti-Aβ (Cell Signaling Technology) and mouse anti-APP (Cell Signaling Technology), then incubated with a species-matched horseradish peroxidase-conjugated secondary antibody. The blots were visualized with a chemiluminescence substrate solution (Pierce) and observed with a chemiluminescent detection system. The optical density of immunoreactive bands was quantified using Quantity One software (Bio-Rad).

For immunoprecipitation, the assay was performed as previously described [47]. Briefly, neurons were lysed with cold RIPA buffer containing a protease inhibitor cocktail and PMSF (Invitrogen). The sample was incubated with 1 μg anti-ANT antibody or 1 μg anti-p-GSK-3β antibody for 2 h at 4 °C. Then, 20 μL of protein A/G plus-agarose slurry was added to the mixture for incubation overnight at 4 °C. The beads were washed 3 times using PBS and boiled in protein sample buffer at 95 °C for 10 min. Equal amounts of proteins were subjected to immunoblot analyses.

### Statistics analysis

All values are represented as mean ± SEM, data analyses were performed using the software of SPSS 19.0. For the Morris water maze tests, escape latency in the hidden platform trial were analyzed with two-way ANOVA of repeated measures, while one-way ANOVA was conducted on the data obtained from the probe trial. A difference was considered significant at *P* < 0.05 level.
